# A Kinase Anchor Protein 4 Is Vulnerable to Oxidative Adduction in Male Germ Cells

**DOI:** 10.3389/fcell.2019.00319

**Published:** 2019-12-20

**Authors:** Brett Nixon, Ilana R. Bernstein, Shenae L. Cafe, Maryse Delehedde, Nicolas Sergeant, Amanda L. Anderson, Natalie A. Trigg, Andrew L. Eamens, Tessa Lord, Matthew D. Dun, Geoffry N. De Iuliis, Elizabeth G. Bromfield

**Affiliations:** ^1^Priority Research Centre for Reproductive Science, School of Environmental and Life Sciences, Discipline of Biological Sciences, The University of Newcastle, Callaghan, NSW, Australia; ^2^Pregnancy and Reproduction Program, Hunter Medical Research Institute, New Lambton Heights, NSW, Australia; ^3^SPQI – 4BioDx-Breeding Section, Lille, France; ^4^University of Lille, INSERM UMRS, Lille, France; ^5^Cancer Signalling Research Group, School of Biomedical Sciences and Pharmacy, Faculty of Health and Medicine, University of Newcastle, Callaghan, NSW, Australia; ^6^Priority Research Centre for Cancer Research Innovation and Translation, Hunter Medical Research Institute, New Lambton Heights, NSW, Australia; ^7^Department of Biochemistry and Cell Biology, Faculty of Veterinary Medicine, Utrecht University, Utrecht, Netherlands

**Keywords:** 4-hydroxynonenal, A-kinase anchor protein 4, male germ cells, oxidative stress, spermatozoa, sperm capacitation, sperm motility

## Abstract

Oxidative stress is a leading causative agent in the defective sperm function associated with male infertility. Such stress commonly manifests via the accumulation of pathological levels of the electrophilic aldehyde, 4-hydroxynonenal (4HNE), generated as a result of lipid peroxidation. This highly reactive lipid aldehyde elicits a spectrum of cytotoxic lesions owing to its propensity to form stable adducts with biomolecules. Notably however, not all elements of the sperm proteome appear to display an equivalent vulnerability to 4HNE modification, with only a small number of putative targets having been identified to date. Here, we validate one such target of 4HNE adduction, A-Kinase Anchor Protein 4 (AKAP4); a major component of the sperm fibrous sheath responsible for regulating the signal transduction and metabolic pathways that support sperm motility and capacitation. Our data confirm that both the precursor (proAKAP4), and mature form of AKAP4, are conserved targets of 4HNE adduction in primary cultures of post-meiotic male germ cells (round spermatids) and in mature mouse and human spermatozoa. We further demonstrate that 4HNE treatment of round spermatids and mature spermatozoa results in a substantial reduction in the levels of both proAKAP4 and AKAP4 proteins. This response proved refractory to pharmacological inhibition of proteolysis, but coincided with an apparent increase in the degree of protein aggregation. Further, we demonstrate that 4HNE-mediated protein degradation and/or aggregation culminates in reduced levels of capacitation-associated phosphorylation in mature human spermatozoa, possibly due to dysregulation of the signaling framework assembled around the AKAP4 scaffold. Together, these findings suggest that AKAP4 plays an important role in the pathophysiological responses to 4HNE, thus strengthening the importance of AKAP4 as a biomarker of sperm quality, and providing the impetus for the design of an efficacious antioxidant-based intervention strategy to alleviate sperm dysfunction.

## Introduction

A male factor is implicated in approximately half of all cases of infertility (Dohle et al., 2005), and in the majority of these individuals, the underlying etiology is attributed to dysregulation of sperm function (Aitken, 2018). Among the myriad of causative agents, oxidative stress has repeatedly been implicated as a key contributor to the loss of sperm developmental and functional competence (Tremellen, 2008; Aitken et al., 2010). Indeed, notwithstanding the important physiological role that reactive oxygen species (ROS) serve to drive the latter phases of sperm maturation (Aitken and Nixon, 2013), exposure to even modest levels of ROS has the potential to induce a state of oxidative stress that deleteriously affects the unique physiology of the male gamete (Aitken et al., 2016). Notably, the legion of pathologic impacts resulting from elevated ROS vary considerably depending on the timing of exposure, with recent evidence suggesting that the round spermatid stage of development is particularly sensitive to oxidative attack. Specifically, post-ROS exposure, round spermatids rapidly succumb to a novel form of regulated cell death, termed ferroptosis (Bromfield et al., 2019). By contrast, equivalent levels of ROS elicit functional lesions in the mature spermatozoon, which compromise their fertilization potential, but does not negatively impact their viability (Bromfield et al., 2015). Such differential pathogenesis may be attributed to the highly specialized architecture of the male germ cell, which depending on their stage of differentiation, features an abundance of substrates for free radical attack, minimal antioxidant defense enzymes, and limited capacity for self-repair when oxidative damage is sustained (Walters et al., 2018b).

Irrespective of the timing of oxidative stress exposure, a virtually ubiquitous response to this insult is lipid peroxidation, a process whereby hydroxyl radicals attack the unsaturated fatty acyl chains of membrane phospholipids (Hauck and Bernlohr, 2016). The ensuing peroxidation reactions generate appreciable levels of highly reactive short chain carbonyl compounds. Among the most abundant and cytotoxic of these secondary oxidation products is 4-hydroxynonenal (4HNE) (Petersen and Doorn, 2004); an aldehyde that has become a major focus in the field due to the pathophysiological impact it exerts on both male (Aitken et al., 2012; Baker et al., 2015; Bromfield et al., 2017b) and female germ cells (Lord et al., 2015; Mihalas et al., 2017, 2018). Under normal physiological conditions, aldehyde-metabolizing enzymes function to detoxify 4HNE, and to abrogate its cellular accumulation, thus limiting its ability to propagate oxidative cellular damage (Hauck and Bernlohr, 2016). However, when challenged with either chronic or acute oxidative stress, 4HNE can accrue within the cellular environment, and, owing to its inherent stability and rapid diffusion, direct the formation of Michael adducts with nucleophilic sites in DNA, lipids and proteins (LoPachin et al., 2009). Curiously, despite 4HNE being capable of disseminating across large functional boundaries, not all elements of the cellular proteome display equivalent susceptibility to 4HNE carbonylation, with only a relatively small number of putative targets identified in spermatozoa to date (Aitken et al., 2012).

In seeking to reconcile the mechanisms underpinning the pathophysiology of 4HNE accumulation, recent studies have reported the application of affinity-based isolation techniques coupled with mass spectrometry to identify 4HNE adducted proteins harbored by oxidatively stressed human spermatozoa (Baker et al., 2015). Among the dominant 4HNE targets identified was A-kinase anchoring protein 4 (AKAP4). AKAP4 is encoded by a single gene located on the X chromosome and synthesized as a precursor pro-polypeptide before undergoing cleavage of the first 188 amino acids to release the mature AKAP4 protein (Carrera et al., 1996; Turner et al., 1998). AKAP4 is subsequently incorporated into the fibrous sheath (Brown et al., 2003); a unique cytoskeletal structure surrounding the axoneme and outer dense fibers that extends throughout the principal-piece of the sperm flagellum (Eddy et al., 2003). Aside from structural contributions to the fibrous sheath, AKAP4 has been implicated as a subcellular scaffold responsible for the tethering of cyclic AMP-dependent protein kinase (PKA), thereby compartmentalizing PKA within the immediate proximity of its enzymatic substrates (Eddy, 2007). Via the targeted positioning of PKA, AKAP4 exerts influence over the specificity of the signal transduction and metabolic processes that support sperm motility and capacitation (Luconi et al., 2011; Sergeant et al., 2019). The importance of AKAP4 in the organization and integrity of the fibrous sheath has been elegantly highlighted by gene ablation studies in which male mice lacking AKAP4 are rendered infertile due to lesions in the progressive motility profiles of their spermatozoa (Miki et al., 2002; Fang et al., 2019). Indeed, while the number of spermatozoa produced by *Akap4* knockout animals remains unchanged, these cells display aberrant fibrous sheath development, a shortened flagella, and a substantially reduced abundance of signal transduction and glycolytic enzymes usually associated with the fibrous sheath (Miki et al., 2002).

These findings take on added significance in view of the dramatic under-representation of AKAP4 in the spermatozoa of infertile human patients (Moretti et al., 2007; Redgrove et al., 2012; Frapsauce et al., 2014). More recent work has also established positive correlations between the levels of AKAP4, and/or that of the proAKAP4 precursor molecule, with key sperm quality and fertility indicators in a number of livestock species (Peddinti et al., 2008; Blommaert et al., 2019; Sergeant et al., 2019). Taken together, these cross species analyses identify the potential use of proAKAP4 and AKAP4 as diagnostic biomarkers of overall semen quality (Sergeant et al., 2019). At present however, it remains uncertain what factor(s) contribute to the striking differences in proAKAP4 and AKAP4 levels documented in livestock (Blommaert et al., 2019) and human spermatozoa (Jumeau et al., 2018). Here, we sought to validate proAKAP4 and AKAP4 as targets for chemical alkylation by 4HNE, and to explore the consequences of 4HNE-mediated alkylation of proAKAP4 and AKAP4 during key phases of sperm development.

## Materials and Methods

### Ethics Statement

All experimental procedures involving animals were conducted with the approval of the University of Newcastle’s Animal Care and Ethics Committee (ACEC) (approval numbers: A-2013–322, A-2018-826). Experiments involving human spermatozoa were performed with semen samples obtained with informed written consent from a panel of healthy normozoospermic donors assembled for the Reproductive Science Group at the University of Newcastle. Volunteer involvement and all experimental procedures were performed in strict accordance with institutional ethics approvals granted by the University of Newcastle Human Research and Ethics Committee (approval number H-2013-0319).

### Reagents

Unless specified, chemical reagents were obtained from Sigma-Aldrich (St. Louis, MO, United States) and were of research grade. Cell culture reagents were purchased from Sigma-Aldrich or Thermo Fisher Scientific (Waltham, MA, United States). The following primary antibodies were used to characterize proteins of interest: monoclonal anti-AKAP4 antibody clone 7E10 (4BDX-1602; 4BioDx, Lille, France), monoclonal anti-proAKAP4 antibody clone 6F12 (4BDX-1701; 4BioDx), rabbit polyclonal anti-4HNE (HNE11-S; Alpha Diagnostic International, San Antonio, TX, United States), rabbit polyclonal anti-androgen receptor (SAB4501575; Sigma-Aldrich), rabbit polyclonal anti-GAPDH antibodies (G9545; Sigma-Aldrich), monoclonal anti-phosphotyrosine (PT66) (P5872; Sigma-Aldrich), rabbit polyclonal anti-phospho (Ser/Thr) PKA substrate (9621; Cell Signaling, Danvers, MA, United States), and rabbit polyclonal anti-amyloid fibrils OC (ab2286; Merck Millipore, Kenilworth, NJ, United States). Appropriate horseradish peroxidase (HRP)-conjugated and Alexa Fluor-conjugated secondary antibodies were obtained from Sigma-Aldrich and Thermo Fisher Scientific, respectively. Bovine serum albumin (BSA) and 3-[(3-cholamidopropyl)dimethylammonio]-1-propanesulfonate (CHAPS) were obtained from Research Organics (Cleveland, OH, United States), Dulbecco’s Modified Eagle Medium (DMEM) was purchased from Thermo Fisher Scientific, Tris was purchased from ICN Biochemicals (Castle Hill, NSW, Australia), nitrocellulose was purchased from GE Healthcare (Buckinghamshire, United Kingdom), Mowiol 4-88 was purchased from Calbiochem (La Jolla, CA, United States), and the paraformaldehyde used in this study was purchased from ProSciTech (Thuringowa, QLD, Australia).

### Mouse Germ Cell Isolation

Swiss mice were obtained from a breeding colony held at the institutes’ central animal house and maintained according to the recommendations prescribed by the ACEC. Mice were housed under a controlled lighting regimen (16L:8D) at 21–22°C and were supplied with food and water *ad libitum*. Prior to dissection, animals were euthanized via CO_2_ inhalation. Enriched populations of spermatocytes and spermatids were isolated from dissected adult mouse testes using density sedimentation at unit gravity as described previously (Nixon et al., 2014; Bromfield et al., 2017b). Briefly, testes were disassociated and tubules were sequentially digested with 0.5 mg/ml collagenase/DMEM and 0.5% (v/v) trypsin/EDTA to remove extra-tubular contents and interstitial cells. The remaining cells were loaded atop a 2–4% (w/v) BSA/DMEM gradient to separate male germ cell types according to their density. Consistent with previous data (Bromfield et al., 2019), this method resulted in the isolation of pachytene spermatocytes (>90% purity) and round spermatids (>85% purity) with minimal somatic cell contamination. Unless stated otherwise, spermatocytes and spermatids were pooled from 2 to 4 mice to achieve adequate cell numbers for subsequent analyses. Experiments were conducted on three independently pooled samples.

### Preparation of Mouse and Human Spermatozoa

Mature spermatozoa were isolated from the cauda epididymides of adult mice by retrograde perfusion and subsequently allowed to disperse into Biggers, Whitten and Whittingham medium (BWW) as previously described (Walsh et al., 2008; Dun et al., 2012). By contrast, corpus spermatozoa were recovered by placing each sampled corpus segment into a 500 μl droplet of modified BWW. After making multiple incisions with a razor blade, the spermatozoa were gently washed into the medium via mild agitation. The resulting suspensions were next placed atop a 27% Percoll density gradient and subjected to centrifugation at 400 × *g* for 15 min at room temperature (RT). The pellet, consisting of an enriched population of >95% corpus spermatozoa was resuspended in fresh BWW and then re-centrifuged at 400 × *g* for 2 min at RT to again pellet the cells and to allow for the removal of excess Percoll (Zhou et al., 2018). These cell preparations were then pooled with those of the cauda epididymis and assessed for viability and motility as previously described (Zhou et al., 2018), prior to being allocated to appropriate treatment groups in preparation for further analysis. In all samples, >80% of the mouse spermatozoa were deemed viable and motile prior to treatment.

Human semen samples were collected following sexual abstinence of at least 2 days. Following collection, all samples were maintained at 37°C and sample analysis was initiated after completion of liquefaction and within 1 h of ejaculation. Each sample was analyzed for total sperm count, motility, morphology and vitality as described previously (Walters et al., 2018a); with all samples used in this study exceeding WHO reference values of 58% live cells, 15 × 10^6^ cells/ml, 1.5 ml ejaculate volume, 40% total motility and 4% normal morphology. After assessment, semen samples were fractionated over a discontinuous Percoll density gradient (comprising 40 and 80% Percoll suspensions) by centrifugation at 500 × *g* for 30 min at RT (Redgrove et al., 2011). This study was restricted to the use of good quality spermatozoa collected from the base of the 80% Percoll suspension as opposed to the poor quality cells partitioning at the 40/80% Percoll interface. After centrifugation, sperm pellets were resuspended in BWW and washed by centrifugation at 500 × *g* for 15 min at RT. Post-washing, the cells were again resuspended in fresh BWW prior to experimental use. A routine assessment of semen parameters was conducted for all donors in accordance with World Health Organization (WHO) criteria and corresponding to the checklist published by Bjorndahl et al. (2016). At least 100 cells were assessed for determination of cell motility, viability and morphology, with at least five microscope fields of view being examined for each count. Sperm morphology was assessed in accordance with WHO criteria using bright field microscopy (×400 magnification; Olympus CX40; Olympus Corporation, Tokyo, Japan). Sperm motility was assessed using phase contrast microscope optics (×400 magnification), with cells being classified as either motile (that is, sperm that displayed any form of motility, ranging from rapid progressive to non-progressive) or immotile.

### 4HNE Treatment

Oxidative stress was induced in germ cells and mature spermatozoa via direct 4HNE (Cayman chemicals, Ann Arbor, MI, United States) challenge, in accordance with our previously established protocols (Bromfield et al., 2015, 2017b; Walters et al., 2018a). For both germ cells and mature spermatozoa, 4HNE treatment regimens (consisting of 4HNE concentrations of either 50 or 100 μM and exposure periods of either 1 or 3 h at 37°C) were selected based on our prior *in vitro* experimentation, in which these conditions were shown to robustly promote the loss and/or dysregulation of alternative 4HNE targeted proteins in male germ cells (Bromfield et al., 2015, 2017a). We also note that the concentrations of 4HNE used herein fall within the range normally associated with low levels of oxidative stress *in vivo* (Uchida, 2003; Chen and Niki, 2006), but are considered sub-lethal such that they are below those required to elicit significant elevation of apoptotic hallmarks (i.e., caspase activation and annexin V binding) or DNA fragmentation (i.e., TUNEL positivity) under comparable *in vitro* incubation periods to those assessed in our study (Aitken et al., 2012). Post-incubation, residual 4HNE was removed via centrifugation at 500 × *g* for 3 min at RT, and resuspension in pre-warmed DMEM or BWW for germ cells and spermatozoa, respectively. Consistent with our previous studies (Bromfield et al., 2015, 2017a; Walters et al., 2018a), the use of these conditions resulted in the death of <20% of the cells under examination (Supplementary Figure S1). In accordance with previous studies (Aitken et al., 2012; Bromfield et al., 2017b, 2019), we also noted that these experimental treatments elicited robust levels of oxidative stress as measured by labeling of the target germ cell populations with MitoSOX Red (MSR) and dihydroethidium (DHE) probes (reference values: ∼40–45% human spermatozoa stained positive for MSR and DHE following 50 μM 4HNE treatment for 3 h). By contrast, equivalent analysis of untreated germ cells confirmed relatively low basal levels of oxidative stress (reference values: <10% human spermatozoa stained positive for MSR and DHE following 50 μM 4HNE treatment for 3 h). Where indicated, an additional control group was incorporated in which male germ cells were pretreated with reduced glutathione (GSH; 4 mM) for 10 min prior to the addition of 4HNE (100 μM). The cells were then co-incubated with both GSH and 4HNE throughout the exposure period (i.e., 3 h for round spermatids and 1 h for spermatozoa). Alternatively, following the completion of 4HNE exposure, the cells were washed twice with BWW (via centrifugation at 500 × *g* for 3 min at RT) to remove residual 4HNE before being resuspended in either DMEM (round spermatids) or BWW (spermatozoa) supplemented with reduced GSH (4 mM) and incubated for an additional 30 min. At the completion of either treatment regimen, gametes were washed twice in BWW via centrifugation at 500 × *g* for 3 min at RT before being processed in accordance with the relevant protocols described below.

### Sodium Dodecyl Sulfate (SDS) Polyacrylamide Gel Electrophoresis and Immunoblotting

Following 4HNE treatment, cells were centrifuged at 500 × *g* for 3 min at RT, and the resulting pellets resuspended in SDS-based protein extraction buffer as previously described (Reid et al., 2012). Protein extracts were then boiled in the presence of NuPAGE LDS sample buffer (Thermo Fisher Scientific) containing 8% β-mercaptoethanol, and subjected to SDS-polyacrylamide gel electrophoresis (SDS-PAGE) using 4–12% Bis–Tris pre-cast gels (Thermo Fisher Scientific). Post-electrophoresis, separated proteins were transferred to nitrocellulose membranes using standard Western blotting techniques (Towbin et al., 1979). To detect proteins of interest, membranes were blocked in 3% BSA (w/v) in Tris-buffered saline supplemented with 0.1% Tween-20 (v/v; TBST, pH 7.4), and then probed with either anti-proAKAP4, anti-AKAP4, anti-4HNE, anti-phospho-PKA substrate, or anti-phosphotyrosine antibodies, as appropriate. All primary antibodies were diluted 1:1000 in TBST supplemented with 1% BSA and probing reactions were conducted overnight at 4°C on a rotating platform. Membranes were next washed in three changes of TBST (10 min/wash) and thereafter, the appropriate secondary antibodies were applied to each membrane for 1 h at RT on a rotating platform. The membranes were again washed with three changes of TBST (10 min/wash), and labeled proteins were then visualized using an enhanced chemiluminescence detection kit according to the manufacturer’s instructions (ECL plus, GE Healthcare). After visualization of the abundance of proteins of interest, all immunoblotted membranes were stripped prior to being re-incubated with anti-GAPDH antibodies (1:4000 dilution), and with the corresponding secondary antibody (washing was performed as outlined above), to demonstrate equivalent protein loading. Band density was quantified in each of three replicate blots using ImageJ software (version 1.48v; National Institute of Health, Bethesda, MD, United States) and the abundance of each protein of interest determined relative to GAPDH labeling intensity. Full length immunoblots of the anti-pro-AKAP4, AKAP4, and GAPDH antibodies used throughout this study are presented in Supplementary Figure S3.

### Immunocytochemistry

Following 4HNE treatment, round spermatids, pachytene spermatocytes, and mature human and mouse spermatozoa, were fixed in 4% paraformaldehyde, washed 3× with 0.05 M glycine in phosphate-buffered saline (PBS). Each cell preparation was then carefully pipetted onto a poly-L-lysine-coated glass coverslip and the cells allowed to settle via overnight incubation at 4°C. Cells were permeabilized with 0.2% (v/v) Triton X-100, then placed in a humidified chamber and blocked with 3% (v/v) BSA/PBS for 1 h at RT. Each coverslip was then washed in PBS and incubated with either anti-proAKAP4, anti-AKAP4, anti-4HNE, anti-phospho-PKA substrate, or anti-phosphotyrosine primary antibodies as appropriate. All primary antibodies were diluted 1:100 in 1% (v/v) BSA/PBS before being applied to coverslips and incubated overnight at 4°C. Coverslips were next washed with three changes of PBS (5 min/wash) before applying the appropriate Alex Fluor-conjugated secondary antibodies (diluted 1:100 in 1% (v/v) BSA/PBS and incubating for 1 h at RT. Coverslips were washed with changes of PBS (3 × 5 min/wash) before mounting in a solution consisting of 10% (v/v) Mowiol 4-88 (Calbiochem) supplemented with 30% (v/v) glycerol in 0.2 M Tris (pH 8.5) and 2.5% (v/v) 1,4-diazabicyclo-(2.2.2)-octane (DABCO). Labeled cells were examined using a Zeiss LSM510 laser scanning confocal microscope (Carl Zeiss).

### Proximity Ligation Assay

Duolink *in situ* primary ligation assays (PLAs) were conducted in accordance with the manufacturers’ instructions (Sigma-Aldrich), on fixed cells adhered to poly-L-lysine-coated coverslips. Briefly, samples were blocked in Duolink blocking solution and then incubated with appropriate primary antibody pairings (anti-proAKAP4, anti-AKAP4, anti-4HNE or anti-amyloid fibrils OC antibodies) overnight at 4°C. Oligonucleotide-conjugated secondary antibodies (PLA probes; anti-rabbit plus, Duo92002; anti-mouse minus Duo82004; Duolink) were then applied for 1 h at 37°C and ligation of the PLA probes was performed. The fluorescent signal generated when molecules are in close association (<40 nm) was visualized using fluorescence microscopy and the number of cells deemed positive for PLA staining was recorded for untreated and 4HNE-treated populations. The specificity of the PLA reaction was ensured by performing proximity ligation with antibodies to the target antigens combined with anti-androgen receptor antibodies with which they should not interact.

### Pharmacological Inhibition of proAKAP4 and AKAP4 Proteolysis

To prevent 4HNE-mediated proteolysis of proAKAP4 and AKAP4 in male germ cells and mature spermatozoa, the cells were treated with several broad-spectrum pharmacological suppressors of proteolytic activity (Complete Mini Protease Inhibitor Cocktail; Roche), or with a selective inhibitor of proteasomal activity (MG132). Alternatively, cells were treated with a selective inhibitor of arachidonate 15-lipoxygenase (PD146176; Tocris Bioscience, Bristol, United Kingdom), an enzyme involved in the propagation of oxidative stress via the metabolism of polyunsaturated fatty acids to 4HNE (Walters et al., 2018b). The concentrations of each inhibitor (Complete Mini Protease Inhibitor Cocktail at 1× working concentration; MG132, 10–25 μM; PD146176, 1 μM), were selected based on published IC_50_ values and from our previous use of these reagents to effectively block the loss of other 4HNE targeted proteins from the germ cell proteome (Bromfield et al., 2017b; Walters et al., 2018a). Germ cells and mature spermatozoa were pre-treated with each inhibitor for 15 min prior to exposure to 4HNE (protease inhibitor cocktail and MG132) or H_2_O_2_ (in the case of PD146176) and the inhibitor was retained for the duration of treatment thereafter to ensure adequate inhibition of each target. A DMSO vehicle control (1 μM) was also included in these experiments.

### Functional Assessment of Human Spermatozoa

Computer Assisted Sperm Analysis (CASA): the movement characteristics of human spermatozoa were assessed using a Hamilton-Thorn motility analyzer (HTMA IVOS II; Hamilton-Thorn Research, Danvers, MA, United States). The settings for human spermatozoa were: 10× NH 160 mm objective, negative phase-contrast optics, recording rate 60 frames/s, minimum head brightness 171, minimum cell size 5 μm^2^, maximum head size 50 μm^2^ and measured in a chamber of 20 μm depth. The criteria of sperm movement assessed were average path velocity (VAP), curvilinear velocity (VCL), straight line velocity (VSL), progressive motility, characterized by a VAP of >25 μm/s and a STR (straightness) of >80%.

Acrosome reaction: Following the induction of capacitation as previously described (Zhou et al., 2017), spermatozoa were induced to acrosome react by supplementation of media with 2.5 μM A23187 for 30 min. The spontaneous rates of acrosome loss were assessed via the inclusion of a capacitated sperm control group, which were prepared under identical incubation conditions with the exception that they did not receive an A23187 stimulus. At the completion of this induction period, the cells were then incubated in pre-warmed hypo-osmotic swelling media (HOS; 0.07% w/v sodium citrate; 1.3% w/v fructose) for another 30 min at 37°C. After being fixed in 4% PFA, spermatozoa were aliquoted onto 12-well slides, air-dried and permeabilized with ice cold methanol for 10 min. Cells were then incubated with fluorescein isothiocyanate (FITC)-conjugated PSA (*Pisum sativum* agglutinin) (1 μg/μl) at 37°C for 15 min, and the acrosomal status of viable cells (possessing coiled tails as a result of incubation in HOS medium) were verified using fluorescence microscopy as previously described (Zhou et al., 2017).

### Statistics

All experiments were replicated at least three times, with each biological replicate comprised of germ cells or mature spermatozoa from at least three mice, or in the case of human spermatozoa, three healthy normozoospermic individuals. Data are expressed as mean values ± SE. Experimental results were analyzed using two-tailed unpaired Student’s *t*-tests (for two way comparison of 4HNE treatments to that of untreated controls) or by one-way analysis of variances (ANOVA) using Microsoft Excel (Version 14.0.0); *post hoc* comparison of group means was by Fisher’s PLSD (protected least significant difference). Differences were considered significant if a *p* < 0.05 was obtained.

## Results

### ProAKAP4 and AKAP4 Are Targeted for 4HNE Adduction in Post-meiotic Male Germ Cells

**FIGURE 1:**
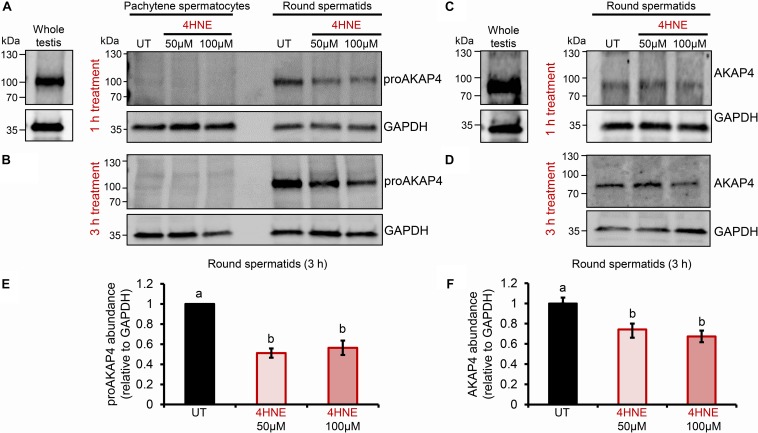
Assessment of the impact of 4HNE treatment on both proAKAP4 and AKAP4 abundance in mouse male germ cells. **(A–D)** Mouse pachytene spermatocytes and round spermatids were exposed to 4-hydroxynonenal (4HNE) (50 or 100 μM) for intervals of either 1 or 3 h before being lysed and subjected to immunoblotting with either **(A,B)** anti-proAKAP4 or **(C,D)** anti-AKAP4 antibodies. Each blot was stripped and re-probed with anti-GAPDH antibodies to confirm equivalent protein loading. **(E,F)** Densitometric analysis of proAKAP4 and AKAP4 band intensity relative to that of GAPDH confirmed significant attenuation of both proteins in 4HNE treated comparted to untreated (UT) round spermatids. Data are expressed as mean ± SEM (*n* = 3). Differing lowercase letters denote statistical significance (*p* < 0.05) as determined by ANOVA.

Previous studies have established that several residues within the AKAP4 primary structure are vulnerable to 4HNE adduction in human spermatozoa. However, the implications of this form of chemical alkylation remain unknown. Here, we therefore, initially assessed the stability of both the AKAP4 and the precursor form, proAKAP4, in mouse germ cell populations treated with exogenous 4HNE (50 or 100 μM) for either 1 or 3 h at 37°C (Figure 1). Consistent with our previous findings (Bromfield et al., 2017a, 2019), the modest level of oxidative stress generated under these exposure regimens led to <20% loss of germ cell viability (Supplementary Figure S1). However, the 1 and 3 h 4HNE treatments did significantly reduce the abundance of both proAKAP4 (Figures 1A,B) and AKAP4 (Figures 1C,D) in post-meiotic round spermatids (*P* < 0.05). Further, the degree of reduction was similar for both the 50 or 100 μM 4HNE treatments (Figures 1E,F). As expected, based on its expression profile (Johnson et al., 1997), the proAKAP4 precursor was not detected in equivalent lysates of untreated pachytene spermatocytes (Figures 1A,B), a finding that confirmed germ cell preparation purity. To account for the possibility of 4HNE-mediated epitope masking, recombinant AKAP4 protein was treated under identical conditions to those imposed on germ cells (i.e., 50 μM 4HNE for 1 h at 37°C) prior to being prepared for immunoblotting. Supplementary Figure S2 clearly demonstrates that 4HNE treated recombinant AKAP4 was detectable at an equivalent level of efficiency to the untreated control with both anti-proAKAP4 and anti-AKAP4 antibodies. Together, these data suggest that the demonstrable reduction in proAKAP4 and AKAP4 immunoreactivity in round spermatids challenged with 4HNE reflects a genuine reduction in the abundance of both precursor and mature forms of AKAP4. This result led us to next attempt to uncover the causative nature of the observed response.

**FIGURE 2:**
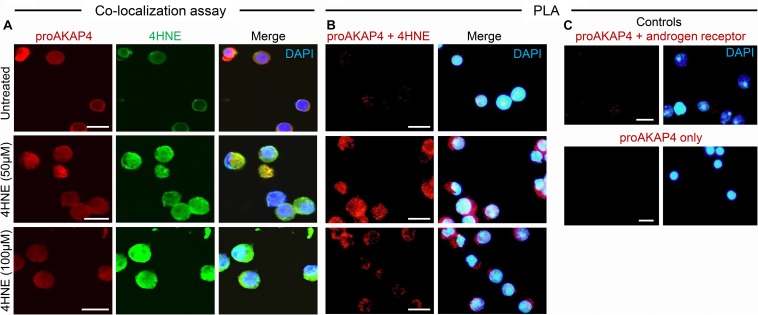
Co-immunolocalization of 4HNE and proAKAP4 in mouse round spermatids. **(A)** Following exposure to 4HNE (50 or 100 μM for 1 h), round spermatids were fixed in paraformaldehyde and sequentially labeled with anti-proAKAP4 (red), anti-4HNE (green) and corresponding fluorescently labeled secondary antibodies. Cells were counterstained with the nuclear stain DAPI (blue). **(B)** Co-localization of target proAKAP4 and 4HNE antigens was confirmed through the application of a proximity ligation assay, whereby fixed round spermatids were incubated with target primary antibodies (anti-proAKAP4 and anti-4HNE) or **(C)** negative controls (anti-proAKAP4 and anti-androgen receptor; anti-proAKAP4 alone) and oligonucleotide-conjugated secondary antibodies (PLA probes). PLA probes were then ligated and the signal was amplified according to the manufacturer’s instructions (Sigma-Aldrich). The red fluorescent signals generated when target antigens reside within <40 nm were visualized using fluorescence microscopy. Scale bars = 10 μm.

Toward this goal, round spermatids were treated with 4HNE prior to assessing the cumulative levels of this aldehyde, and the extent of 4HNE co-localization with the proAKAP4 precursor; the isoform determined most affected by 4HNE treatment (Figure 1E). Consistent with the immunoblotting data, the accumulation of 4HNE throughout the cytosol of round spermatids was accompanied by a reciprocal reduction in the amount of immunoreactive proAKAP4 detected within these cells (Figure 2A). In addition, the residual proAKAP4 remaining in round spermatids appeared to co-localize with 4HNE labeling (Figure 2A). This co-localized accumulation profile for 4HNE and proAKAP4, was confirmed via use of the proximity ligation assay, which revealed intense labeling foci for the paired proAKAP4 and 4HNE antibodies throughout the round spermatid cytosol (Figure 2B). Notwithstanding the basal levels of 4HNE detected in untreated round spermatids via conventional immunolabeling (Figure 2A), this naïve cell population was characterized by very few PLA positive foci when assessed using the same antibody pairing (anti-proAKAP4 and anti-4HNE) (Figure 2B). The specificity of PLA labeling was confirmed via inclusion of two controls, namely; antibodies against the androgen receptor protein that is not known to interact with proAKAP4, as well as the use of a single antibody only (anti-proAKAP4) control. Figure 2C clearly shows that each control failed to generate a positive PLA signal (red fluorescence). This result confirms the close proximity of 4HNE to the proAKAP4 precursor in the cytosol of round spermatids.

**FIGURE 3:**
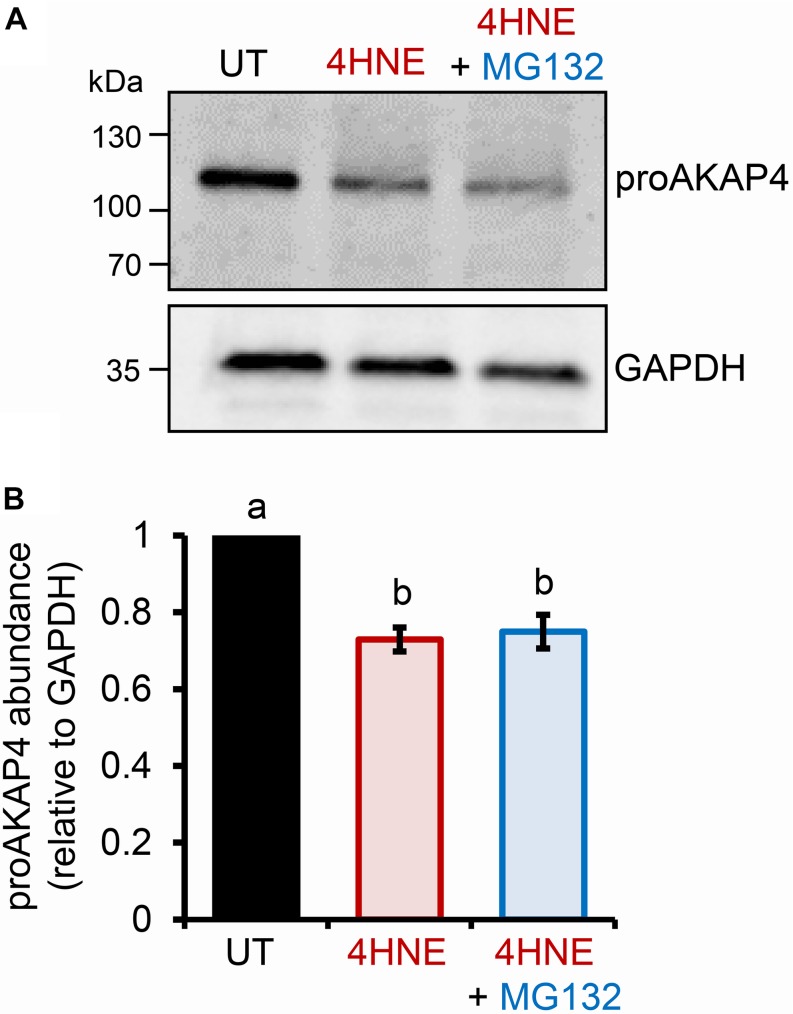
Inhibition of proteasome activity does not rescue 4HNE mediated loss of proAKAP4 in round spermatids. **(A)** Round spermatids were pre-treated with MG132 (proteasome inhibitor) for 15 min prior to exposure to 4HNE (100 μM, 3 h at 37°C), with the inhibitor being retained for the duration of treatment. Cells were solubilized in an SDS based extraction buffer prior to being resolved by SDS-PAGE and prepared for immunoblotting with anti-proAKAP4 antibodies. Blots were subsequently stripped and reprobed with anti-GAPDH antibodies to ensure equivalent protein loading. **(B)** Labeled bands corresponding to proteins of interest were subjected to band densitometry analysis and mean values (±SEM) are presented relative to the corresponding GAPDH control (*n* = 3). Differing lowercase letters denote statistical significance (*p* < 0.05) as determined by ANOVA. UT = untreated control.

The preceding data strongly suggests that elevated levels of intracellular 4HNE leads to proAKAP4 adduction, possibly destabilizing the structure of the precursor; a modification that would likely interfere with the formation of mature AKAP4. The ubiquitin-proteasome pathway has been previously implicated in the selective elimination of a variety of oxidatively damaged proteins (Davies, 2001; Shringarpure et al., 2001). We therefore, next assessed the contribution of the proteasomal pathway to the clearance of modified proAKAP4. For this purpose, round spermatids were co-incubated with 4HNE in tandem with MG132, a cell-permeable synthetic peptide that is widely used to repress proteasome activity. Unexpectedly however, MG132 co-incubation failed to rescue 4HNE-mediated loss of proAKAP4 in round spermatids (Figures 3A,B). Similarly, co-incubation with a broad-spectrum protease inhibitor cocktail also had minimal effect on the abundance of proAKAP4 in 4HNE-treated round spermatids, when used either alone, or in tandem with MG132 (data not shown). This result led to the conclusion that 4HNE-mediated loss of proAKAP4 in round spermatids is unlikely to involve conventional proteolytic degradation.

### Acute 4HNE Exposure Reduces proAKAP4 and AKAP4 Abundance in Mouse and Human Spermatozoa

**FIGURE 4:**
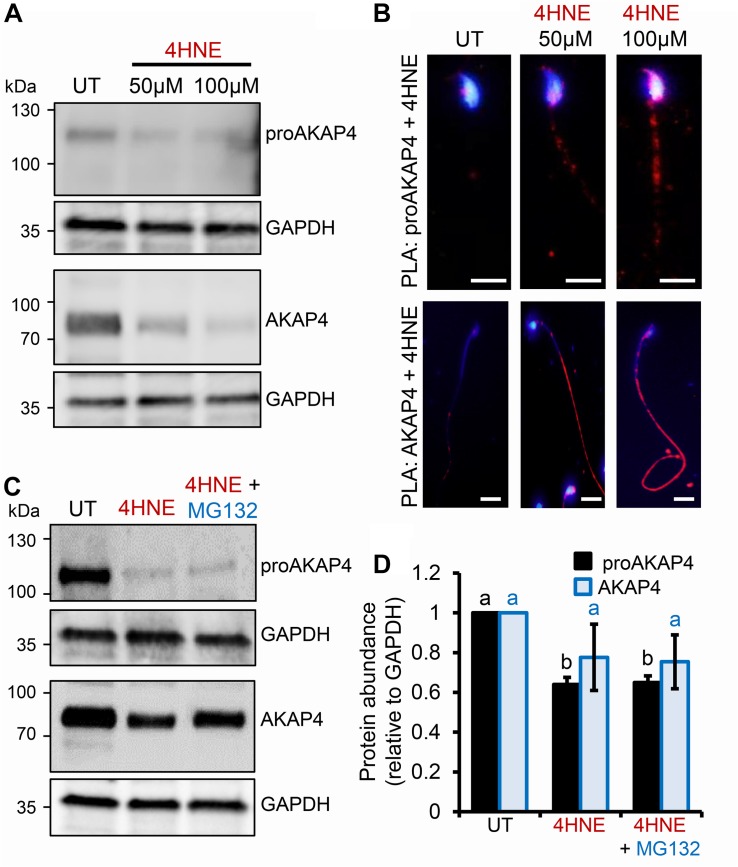
Assessment of 4HNE adduction to proAKAP4 and AKAP4 in mature mouse spermatozoa. **(A,B)** Mouse spermatozoa were exposed to 4HNE (50 or 100 μM) alone or **(C,D)** in the presence of the proteasome inhibitor MG132 and incubated for 1 h at 37°C. **(A,C)** Spermatozoa were prepared for immunoblotting analysis with anti-proAKAP4 and anti-AKAP4 antibodies. Each blot was stripped and re-probed with anti-GAPDH antibodies to confirm equivalent protein loading. **(B)** The adduction of proAKAP4 and AKAP4 with 4HNE was assessed through the application of a proximity ligation assay, whereby fixed spermatozoa were incubated with target primary antibodies (anti-proAKAP4 or anti-AKAP4 and anti-4HNE) or **(C)** negative controls (anti-proAKAP4 and anti-AKAP4 and anti-androgen receptor; anti-proAKAP4 alone) and oligonucleotide-conjugated secondary antibodies (PLA probes). PLA probes were then ligated and the signal was amplified. The red fluorescent signals generated when target antigens reside within <40 nm were visualized using fluorescence microscopy and representative images of each treatment group are presented. **(D)** Protein abundance was quantified by band densitometry and mean values (±SEM) are presented relative to the corresponding GAPDH control (*n* = 3). Differing lowercase letters denote statistical significance (*p* < 0.05) as determined by ANOVA. Scale bars = 5 μm. UT = untreated control.

Given the pronounced 4HNE-mediated loss of proAKAP4 and AKAP4 in post-meiotic round spermatids, we next extended our analyses to assess the consequences of 4HNE treatment of mouse and human spermatozoa; terminally differentiated cells that have a reduced capacity to ameliorate the deleterious effects of oxidative insult compared to that of precursor germ cells. In mouse spermatozoa, 4HNE treatment reduced the abundance of both proAKAP4 and AKAP4 (Figures 4A,D). However, proAKAP4 appeared more sensitive in these cells, experiencing a highly significant ∼40% reduction (*P* < 0.01), compared to the untreated controls (Figures 4A,D). Consistent with our data on the round spermatid (Figure 2), the application of PLA revealed intense co-labeling of 4HNE with proAKAP4 and AKAP4 within the expected domains of the sperm flagellum (Figure 4B). More specifically, PLA fluorescence was restricted to the proximal portion of the sperm flagellum for the 4HNE and proAKAP4 antibody pairing, whilst the 4HNE and AKAP4 pairing allowed for the visualization of fluorescent foci throughout the entire principal piece (Figure 4B). Together, these data indicate that residual proAKAP4 and AKAP4 proteins that remain within the flagellum of 4HNE treated mouse spermatozoa likely harbor substantial levels of aldehyde adducts. It is also important to note here that, as observed for 4HNE-treated round spermatids, co-incubation with the proteasome inhibitor, MG132, failed to rescue the loss of either proAKAP4 or AKAP4 in 4HNE-treated mature mouse spermatozoa (Figures 4C,D).

**FIGURE 5:**
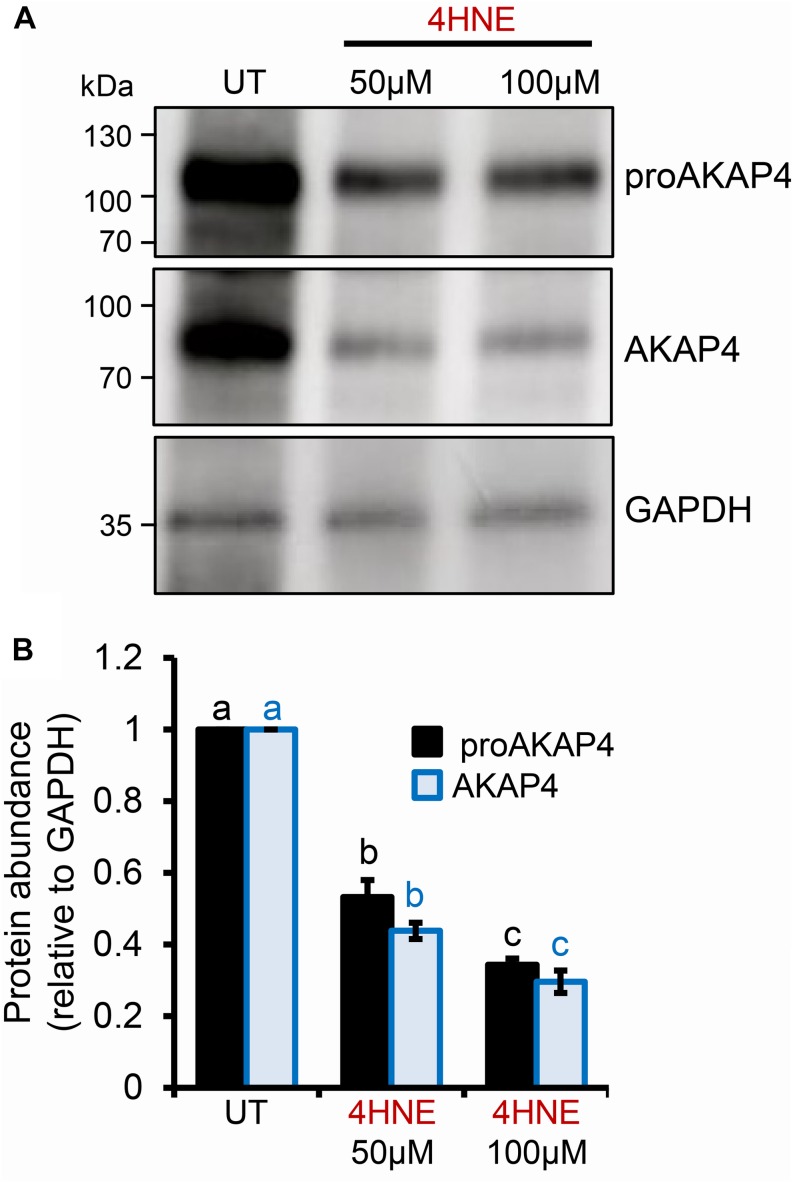
Assessment of the impact of 4HNE adduction on proAKAP4/AKAP4 protein abundance in mature human spermatozoa. **(A)** Human spermatozoa were incubated with 4HNE (50 or 100 μM) for 1 h at 37°C to induce oxidative stress. Cells were subsequently prepared for immunoblotting analysis with anti-proAKAP4 and anti-AKAP4 antibodies. Each blot was stripped and re-probed with anti-GAPDH antibodies to confirm equivalent protein loading. This analysis was performed in triplicate using spermatozoa from three different donors and a representative blot is depicted. **(B)** Protein abundance was quantified by band densitometry and mean values (±SEM) are presented relative to the corresponding GAPDH control (*n* = 3). Differing lowercase letters denote statistical significance (*p* < 0.05) as determined by ANOVA. UT = untreated control.

**FIGURE 6:**
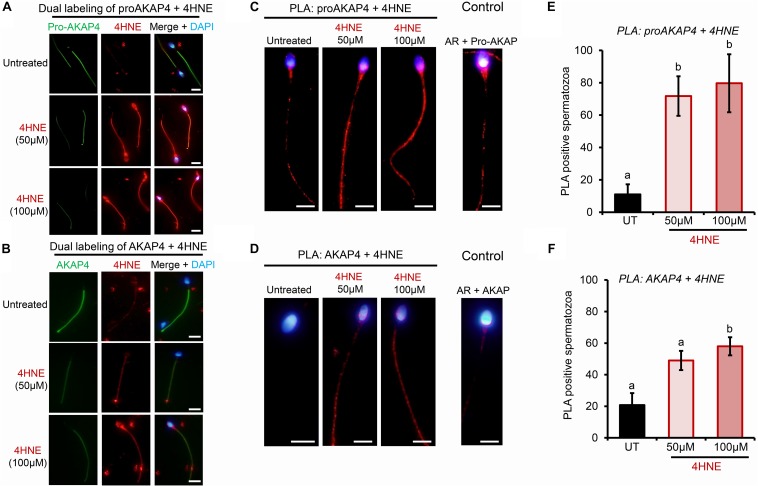
Confirmation of 4HNE adduction to proAKAP4 and AKAP4 in mature human spermatozoa. Human spermatozoa were incubated with 4HNE (50 or 100 μM) for 1 h to induce oxidative stress before being fixed with paraformaldehyde. **(A,B)** Co-localization of proAKAP4 and AKAP4 with 4HNE was performed by dual labeling of spermatozoa with anti-proAKAP4, anti-AKAP4 and anti-4HNE primary antibodies, followed by incubation with corresponding Alexa Fluor conjugated secondary antibodies. This protocol was repeated in the absence of either primary antibody to generate a secondary antibody only control. Representative images were captured using fluorescence microscopy. **(C,D)** The adduction of proAKAP4 and AKAP4 with 4HNE was assessed through the application of a proximity ligation assay, whereby fixed spermatozoa were incubated with target primary antibodies (anti-proAKAP4 or anti-AKAP4 and anti-4HNE) or negative controls [anti-proAKAP4 or anti-AKAP4 and anti-androgen receptor (AR)] and oligonucleotide-conjugated secondary antibodies (PLA probes). PLA probes were then ligated and the signal was amplified. The red fluorescent PLA signals were visualized using fluorescence microscopy and representative images of each treatment group are presented. **(E,F)** Positive proAKAP4/AKAP4 and 4HNE interaction was assessed in a minimum of 100 cells per treatment group using fluorescence microscopy and the percentage of PLA positive cells is expressed as mean values ± SEM (*n* = 3). Differing lowercase letters denote statistical significance (*p* < 0.05) as determined by ANOVA. Scale bars = 5 μm. UT = untreated control.

To determine if AKAP4 is similarly targeted for 4HNE-mediated adduction in human spermatozoa, these cells were treated as per their mouse counterparts. Initially, proAKAP4 and AKAP4 abundance was assessed in the spermatozoa of three healthy normozoospermic donors, with each sample subjected to acute 4HNE treatment (50 or 100 μM) for 1 h at 37°C. As shown in the representative immunoblots in Figure 5A, sperm from each donor responded to 4HNE challenge in an equivalent manner, that is; proAKAP4 and AKAP4 abundance was significantly reduced in a dose-dependent manner (*p* < 0.01). For the 100 μM 4HNE treatment, both the precursor and mature form of AKAP4 were reduced by as much as 70% compared to the untreated control samples (Figure 5B). Accordingly, immunocytochemistry revealed elevated levels of 4HNE and a concomitant reduction in proAKAP4 and AKAP4 abundance within the flagellum of each 4HNE-treated human sperm sample assessed (Figures 6A,B). Furthermore, PLA fluorescence was observed in the majority of these cells post-4HNE treatment when the 4HNE antibody was paired with either the proAKAP4 or AKAP4 antibody (Figures 6C–F). Although basal levels of PLA fluorescence were detected in untreated control spermatozoa, both the distribution and intensity of this labeling appeared equivalent to that detected in the irrelevant antibody control samples, suggesting that the detected signal was non-specific in nature (Figures 6C,D). Overall, these data are in general agreement with that generated for mouse spermatozoa (Figure 4), thus indicating cross-species conservation in terms of the vulnerability of both the precursor and mature form of AKAP4 to 4HNE insult as well as the fate of the alkylated proteins.

### Consequences of 4HNE-Adduction of ProAKAP4 and AKAP4

**FIGURE 7:**
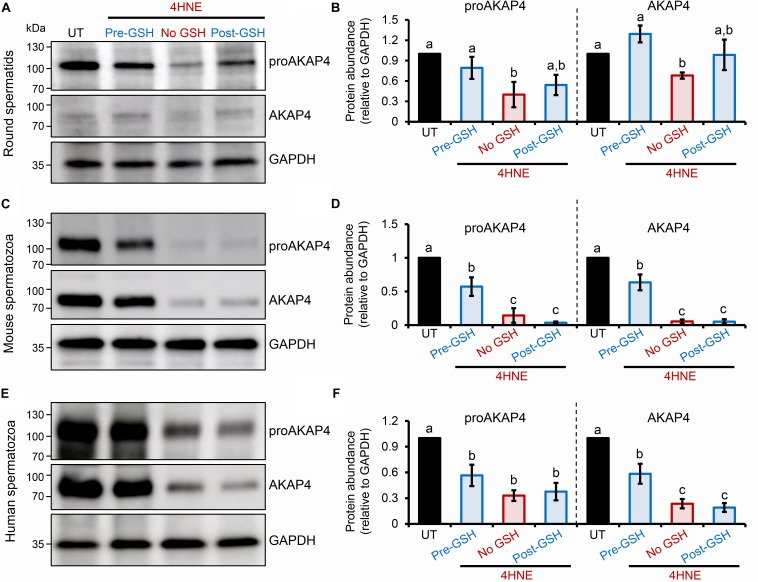
Assessment of the impact of GSH on proAKAP4 and AKAP4 abundance in populations of 4HNE-treated round spermatids and mature spermatozoa. **(A,B)** Round spermatids, and mature **(C,D)** mouse and **(E,F)** human spermatozoa were pre-treated with reduced glutathione (GSH; 4 mM) for 10 min prior to the addition of 4HNE (100 μM). The cells were then co-incubated with both GSH and 4HNE throughout the exposure period (Pre-GSH). Alternatively, following the completion of 4HNE exposure, the cells were washed to remove residual 4HNE before resuspended in media supplemented with GSH (4 mM) and incubated for an additional 30 min (Post-GSH). At the completion of either treatment regimen, gametes were washed before being processed for immunoblotting with either anti-proAKAP4 or anti-AKAP4 antibodies. Each blot was stripped and re-probed with anti-GAPDH antibodies to confirm equivalent protein loading. This analysis was performed in triplicate and representative blots are depicted. **(B,D,F)** Protein abundance was quantified by band densitometry and mean values (±SEM) are presented relative to the corresponding GAPDH control (*n* = 3). Differing lowercase letters denote statistical significance (*p* < 0.05) as determined by ANOVA. UT = untreated control.

**FIGURE 8:**
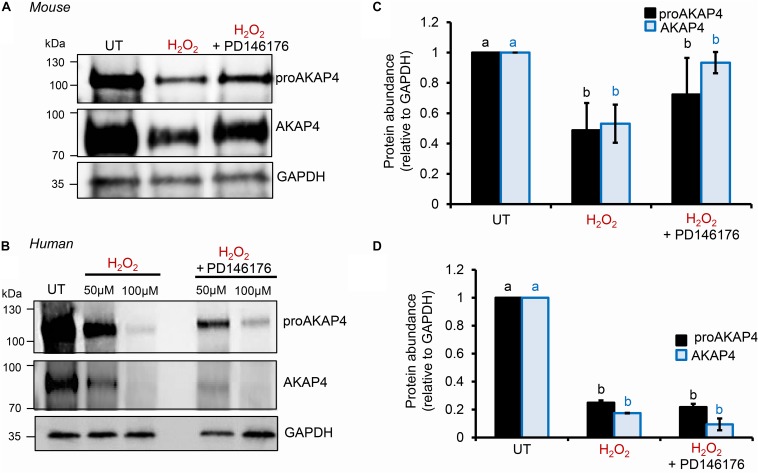
Assessment of ALOX15 inhibition on proAKAP4 and AKAP4 in mouse and human spermatozoa. **(A,B)** Mouse and human sperm samples were pre-treated with PD146176 to inhibit ALOX15 (an enzyme implicated in driving a positive feedback loop of lipid peroxidation) for 15 min prior to exposure to H_2_O_2_ for 1 h at 37°C, with the inhibitor being retained for the duration of treatment. A DMSO vehicle control was also included in experiments. Sperm lysates were prepared for immunoblotting with anti-proAKAP4 and anti-AKAP4 antibodies. Blots were subsequently stripped and reprobed with anti-GAPDH antibodies to ensure equivalent protein loading. All **(A)** mouse and **(B)** human protein lysates were resolved on the same gels but have been cut and spliced for presentation purposes. **(C,D)** Labeled bands corresponding to proteins of interest were subjected to band densitometry analysis and mean values (±SEM) are presented expressed relative to the corresponding GAPDH control (*n* = 3). Differing lowercase letters denote statistical significance (*p* < 0.05) as determined by ANOVA. UT = untreated control.

To explore the causal nature of 4HNE-mediated attenuation of proAKAP4 and AKAP4 abundance, populations of round spermatids, mouse and human spermatozoa were treated with reduced glutathione (GSH) prior to, or after, implementing the 4HNE exposure. As anticipated based on the ability of GSH to directly alkylate and thereby reduce the bioavailability of 4HNE, pre-incubation of male gametes with this antioxidant conferred at least some degree of protection to proAKAP4 and AKAP4 proteins. Indeed, the abundance of both proteins detected in pre-GSH treated round spermatids was statistically indistinguishable from that of the untreated control samples. An equivalent trend was observed in mouse and human spermatozoa, although in both instances the abundance of proAKAP4 and AKAP4 proteins was still reduced in pre-GSH treated cells versus that of their untreated counterparts. In terms of the response to the opposing post-GSH treatment, this again varied depending on the cell type analyzed. Thus, in the case of round spermatids, which retain some capacity for protein synthesis, proAKAP4 and AKAP4 levels were recovered to levels that proved statistically similar to that of untreated controls (Figures 7A,B). However, the retrospective application of GSH failed to rescue either proAKAP4 or AKAP4 levels in 4HNE treated mouse or human spermatozoa (Figures 7C–F). These results prompted us to explore whether alternative strategies could be used to prevent 4HNE-mediated reduction of proAKAP4 and AKAP4 in mature spermatozoa. With this goal in mind, we next investigated whether the abundance of the two AKAP4 isoforms could be maintained via the administration of a pharmacological inhibitor that acts upstream of 4HNE production. Specifically, spermatozoa were subjected to oxidative stress in the form of an H_2_O_2_ insult either alone, or in the presence of PD146176, a selective inhibitor of the lipoxygenase enzyme, arachidonate 15-lipoxygenase (ALOX15); an enzyme that drives a positive feedback loop of lipid peroxidation leading to amplification of 4HNE generation in male germ cells (Walters et al., 2018a). Accordingly, via the administration of H_2_O_2_ alone, we were able to reproduce the previously observed reduction in the abundance of both proAKAP4 and AKAP4 from mouse and human spermatozoa (Figures 8A–D). Further, in H_2_O_2_ alone treated samples, proAKAP4 and AKAP4 were reduced to levels similar to those elicited by the direct application of 4HNE. Unexpectedly however, co-incubation of PD146176 together with H_2_O_2_, led to only a partial recovery of proAKAP4 and AKAP4 levels in mouse spermatozoa (Figures 8A,C), and proved completely ineffective in the rescue of proAKAP4 and AKAP4 in human spermatozoa (Figures 8B,D).

**FIGURE 9:**
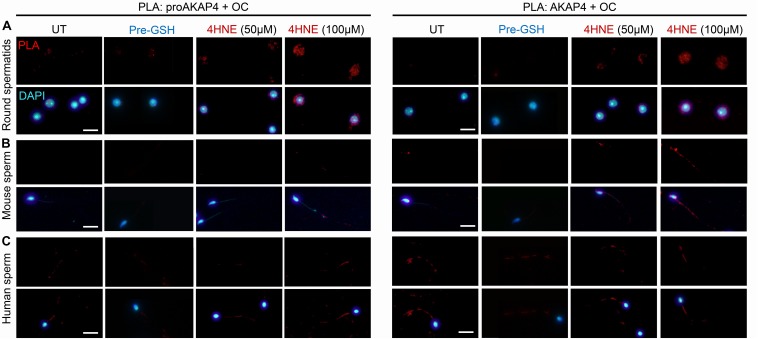
Assessment of the aggregation potential of 4HNE modified proAKAP4 and AKAP4 in male germ cells. Populations of **(A)** round spermatids, **(B)** mouse spermatozoa, and **(C)** human spermatozoa were incubated with 4HNE (50 or 100 μM) for 1 h to induce oxidative stress before being fixed with paraformaldehyde. The propensity of 4HNE adducted proAKAP4 and AKAP4 to form aggregates was assessed through the application of PLA, whereby fixed spermatozoa were incubated with target primary antibodies (anti-proAKAP4 or anti-AKAP4 and anti-amyloid fibrils OC antibodies) and oligonucleotide-conjugated secondary antibodies (PLA probes). In addition to an untreated control (UT), gametes were also pre-treated with reduced glutathione (GSH; 4 mM) for 10 min prior to the addition of 4HNE (100 μM). The cells were then co-incubated with both GSH and 4HNE throughout the exposure period (Pre-GSH). PLA fluorescent signals were visualized using fluorescence microscopy and representative images of each treatment group are presented. This experiment was replicated three times and representative images are depicted. Scale bars = 10 μm.

In view of these data, we next attempted to determine if 4HNE adduction can act as a catalyst to destabilize either the precursor or mature form of AKAP4, in order to drive the protein toward aggregate formation. Thus, PLA was again employed to assess the dual labeling of either the proAKAP4 or AKAP4 antibody together with an amyloid fibrils OC-specific antibody (Figure 8). The amyloid fibril OC antibody was selected for inclusion in this analysis based on its recognition of generic epitopes common to many amyloid fibrils and fibrillary oligomers, but not prefibrillar oligomers or natively folded precursors. As shown in Figure 9A, PLA fluorescence was observed for both antibody pairings throughout the cytosol of 4HNE-treated round spermatids, with the labeling appearing more intense at the higher 4HNE concentration of 100 μM (Figure 9A). PLA fluorescence, albeit less intense, was also observed for both antibody pairings within the flagellum of 4HNE-treated mouse (Figure 9B) and human spermatozoa (Figure 9C). In all instances, PLA labeling was attenuated via prior incubation of the germ cells with GSH before they received 4HNE-exposure (Figures 9A–C). Taken together, these data suggest that in male germ cells, 4HNE adduction can result in the aggregation of both the precursor and mature forms of AKAP4.

**FIGURE 10:**
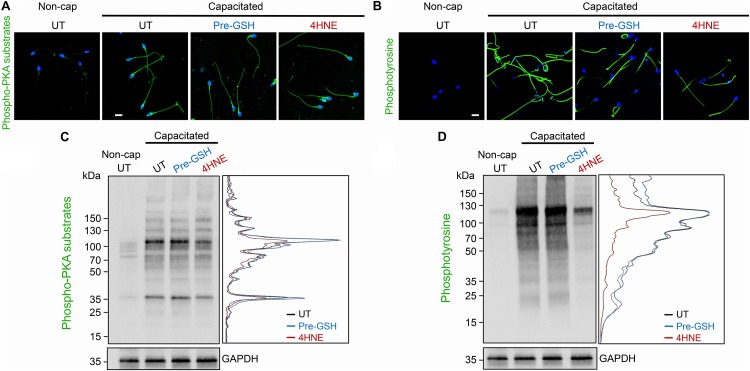
4HNE adduction of proAKAP4 and AKAP4 attenuates capacitation-associated signaling in human spermatozoa. Human spermatozoa treated with 4HNE (100 μM for 1 h at 37°C) were induced to capacitate (Capacitated) or held in a non-capacitated state (Non-cap) using standard protocols (Mitchell et al., 2007). Spermatozoa were subsequently prepared for either **(A,B)** immunocytochemistry or **(C,D)** immunoblotting with anti-phospho-PKA substrate or anti-phosphotyrosine antibodies. A densitometric trace of representative immunoblots is included to highlight changes in phospho-labeling in untreated (UT; black trace), GSH pre-treated (Pre-GSH; blue trace) and 4HNE treated (4HNE; red trace) spermatozoa. This experiment was replicated with samples from three separate donors and representative images are immunoblots are presented. Scale bars = 10 μm.

A potential consequence of 4HNE-mediated protein degradation and/or aggregation is dysregulation of the cAMP signaling framework assembled around the AKAP4 scaffold in mature spermatozoa. To address this possibility, human spermatozoa were subjected to 4HNE treatment before being driven to capacitate and assessed for the phosphorylation of PKA and tyrosine kinase substrates. Immunocytochemical analysis demonstrated that our 4HNE treatment regimen did not overtly impact the localization of proteins being phosphorylated during the capacitation of human spermatozoa, with prominent staining being evident throughout the sperm flagellum (Figures 10A,B). However, immunoblotting confirmed that 4HNE treatment did in fact lead to reduced levels of phosphorylation among a portion of the intermediary PKA targets, particularly the prominent bands resolving at ∼35 and 100 kDa (Figure 10C). In addition, 4HNE elicited a more pronounced, global reduction in the phosphorylation levels of downstream tyrosine kinase substrates (Figure 10D). In both instances, these effects were reduced by prior incubation of the human spermatozoa with reduced GSH before the addition of 4HNE (Figures 10A–D).

**FIGURE 11:**
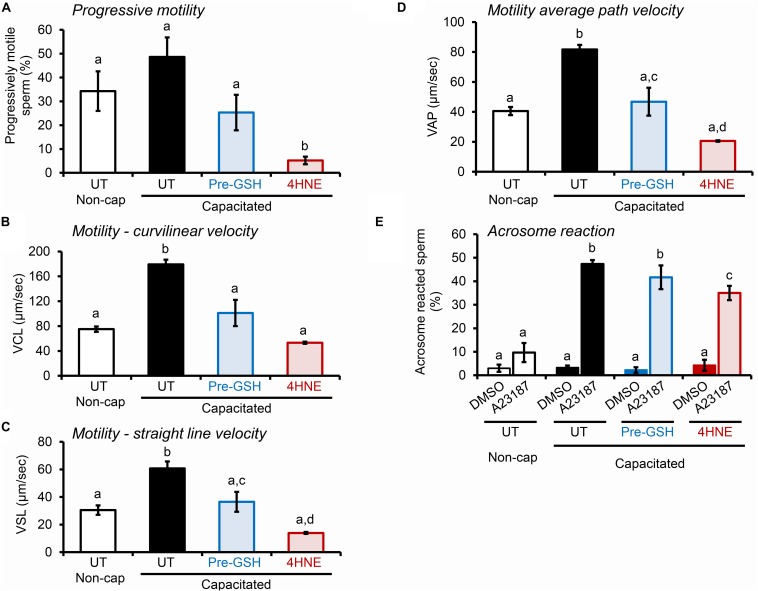
4HNE negatively impacts the motility profile and ability of human spermatozoa to complete an acrosome reaction. **(A–D)** Human spermatozoa treated with 4HNE (100 μM for 1 h at 37°C) were induced to capacitate (Capacitated) or held in a non-capacitated state (Non-cap) using standard protocols (Mitchell et al., 2007). Computer Assisted Sperm Analysis (CASA) was subsequently used to objectively track the movement characteristics of human spermatozoa and the assessment criteria of **(A)** progressive motility, **(B)** curvilinear velocity (VCL), **(C)** straight line velocity (VSL), and **(D)** average path velocity (VAP) are presented. In addition to an untreated control (UT), spermatozoa were also pre-treated with reduced glutathione (GSH; 4 mM) for 10 min prior to the addition of 4HNE and the cells were then co-incubated with both GSH and 4HNE throughout the exposure period (Pre-GSH). **(E)** Alternatively, the spermatozoa were assessed for their competence to complete a calcium ionophore (A23187) induced acrosome reaction, via staining with fluorescently labeled PSA as previously described (Zhou et al., 2017). The spontaneous rates of acrosome loss were assessed via the inclusion of a vehicle control group (DMSO). These experiments were replicated with samples from three separate donors and representative images are and mean values (±SEM) are presented. Differing lowercase letters denote statistical significance (*p* < 0.05) as determined by ANOVA.

Notably, the deleterious impact of 4HNE extended to alterations in the motility profile of capacitated human spermatozoa (Figures 11A–D), as well as the ability of these cells to undergo acrosomal exocytosis (Figure 11E). Interestingly in this regard, in addition to a subtle, albeit significant reduction in the proportion of human spermatozoa exhibiting some form of motility (i.e., total motile sperm count; Supplementary Figure S1E), the assessment of key parameters of sperm movement, including average path velocity, curvilinear velocity, straight line velocity, and progressive motility, were all significantly attenuated after incubation with 4HNE (Figures 11A–D). Similarly, human spermatozoa experienced a significant, ∼25% reduction in their ability to complete a calcium ionophore (A23187) induced acrosome reaction (Figure 11E). Once again, these damaging effects of 4HNE were ameliorated via pre-treatment of spermatozoa with GSH.

## Discussion

Driven by unprecedented rates of recourse to assisted reproductive technologies, there remains a pressing need to develop robust biomarkers capable of predicting fertilization success and discriminating the causative agents that can be targeted to prevent reproductive failure (Aitken et al., 2010). Consistent with these objectives, here, we examined the effects of sub-lethal doses of oxidative insult on the expression of proAKAP4 and of AKAP4; proteins that hold pivotal roles in the formation and structure of the sperm flagellar, in addition to coordination of capacitation-associated signaling (Luconi et al., 2011). We demonstrate that both proAKAP4 and AKAP4 are highly sensitive to oxidative challenge throughout the development of the male germ cell. Indeed, exposure to the lipid aldehyde, 4HNE, led to a substantive loss of both proAKAP4 and AKAP4 in round spermatids and mature spermatozoa. In addition, the residual proAKAP4 and AKAP4 that remained post-4HNE treatment were demonstrated to harbor the burden of 4HNE chemical alkylation; a modification that may perturb the stability of the protein native state, resulting in misfolding and nucleation of protein aggregates as has been shown in somatic cells (Maniti et al., 2015).

As the focus of this study, AKAP4 is a member of a large family of structurally diverse, but functionally conserved, anchoring proteins that coordinate the subcellular distribution of cAMP-mediated signaling networks (Colledge and Scott, 1999). Accordingly, a defining feature of the AKAP family is the presence of at least one PKA anchoring domain that serves to position the PKA holoenzyme at locations where it can rapidly respond to fluctuations in cAMP production. However, the multivalent nature of the AKAP scaffold has also implicated this protein in the physical tethering of several kinases, phosphatases, ion channels, and GTP binding proteins (Colledge and Scott, 1999). This property enables AKAPs to orchestrate the formation of macromolecular complexes compatible with the integration of cAMP and alternate intracellular signaling networks (Colledge and Scott, 1999). In the context of spermatozoa, the compartmentalization of cAMP signaling machinery coordinated by AKAP4 serves to regulate the specificity of signal transduction pathways responsible for the development, activation and maintenance of motility (Luconi et al., 2011; Sergeant et al., 2019). In accordance with this fundamental role, AKAP4 expression displays high evolutionary conservation, with *Akap4* transcripts and/or immunoreactive orthologs of AKAP4 having been characterized in the testes and spermatozoa of species as phylogenetically diverse as eutherian mammals (humans, rodents, bovine, equine, porcine) (Johnson et al., 1997; Turner et al., 1998; Moss et al., 1999; Teijeiro and Marini, 2012; Blommaert et al., 2019; Sergeant et al., 2019), marsupials (tammar wallaby, opossum) (Hu et al., 2009), monotremes (platypus) (Hu et al., 2009), and reptiles (crocodile) (Nixon et al., 2019).

In the mouse testes, AKAP4 is synthesized as a precursor of ∼100 kDa (proAKAP4) during the post-meiotic phase of spermatogenesis, with transcripts first detected in early stage round spermatids (Johnson et al., 1997; Brown et al., 2003). Following synthesis, proAKAP4 is processed via the proteolytic cleavage of an N-terminal prodomain, yielding the mature AKAP4 protein (∼82 kDa) (Carrera et al., 1994). AKAP4 is subsequently transported to the principal piece of developing flagellum to become the most abundant structural element of the nascent fibrous sheath; accounting for as much 50% of the protein within this specialized domain. The fate of proAKAP4 is somewhat less certain, with emerging evidence supporting interspecies differences in both the extent of its processing and distribution within the mature sperm flagellum. Thus, proAKAP4 has been reported to localize along the entire length of the principal piece of mouse testicular spermatozoa before becoming restricted to the proximal portion of the flagellum in mature cauda epididymal spermatozoa (Johnson et al., 1997); suggestive of an additional wave of proAKAP4 processing during epididymal sperm maturation. By contrast, and in agreement with our own data, proAKAP4 is retained within the flagellum of mature human spermatozoa, where it co-localizes with AKAP4 throughout the fibrous sheath (Turner et al., 1998; Jumeau et al., 2018). Notably however, striking differences in proAKAP4 abundance have been documented in the semen of normozoospermic individuals, wherein the quantity of the protein is positively correlated with sperm motility; albeit in the high-quality cells recovered by density gradient centrifugation (Jumeau et al., 2018). Whilst these data invite speculation that the extent of proAKAP4 processing may reflect the integrity of post-testicular sperm maturation, and hence contribute to differences in the motility profile of human spermatozoa, there is currently no causal evidence to substantiate such an association. Rather, it has been postulated that proAKAP4 may serve as a “reservoir” that can be activated to generate mature AKAP4 and thus rescue sperm motility, if and when required (Jumeau et al., 2018; Sergeant et al., 2019).

Our collective findings confirm previous work in identifying AKAP4 as a primary target of 4HNE adduction in mature human spermatozoa. Indeed, studies by Baker et al. (2015) identified at least three AKAP4 peptides that harbor either one or two 4HNE modified residues after exogenous treatment of spermatozoa with the aldehyde. This study not only confirmed a significant 11-fold enrichment in modified AKAP4 peptides isolated after 4HNE treatment, but also revealed basal levels of endogenous 4HNE modification of AKAP4 in untreated spermatozoa (Baker et al., 2015); data that further reinforces the inherent sensitivity of AKAP4 to chemical alkylation reactions. Though the full extent of AKAP4 damage elicited by insertion of bulky 4HNE (C-9) carbonyl adducts has yet to be investigated, our *in silico* modeling of 4HNE modified residues (K_279_, K_331_, C_566_ and C_570_), indicates that these lie outside of the prodomain (M_1_-N_188_) and those regions implicated in the binding of PKA regulatory subunits (i.e., F_219_–A_232_ and I_336_–K_345_) (Miki and Eddy, 1998, 1999). The 4HNE modified residues do however, reside close to several putative phosphorylation sites and include cysteine residues that may be involved in stabilization of AKAP4 tertiary structure. By analogy with other 4HNE targets, it is reasonable to suspect that these modifications could elicit protein mis-folding, poor substrate recognition, and/or degradation of the protein itself (Carbone et al., 2004a, b); lesions that likely contribute to the dysregulation of sperm motility witnessed in this study as well as that commonly reported in cells burdened by excessive ROS production (Aitken et al., 2010).

In extrapolating these data beyond motility, the importance of the signaling framework coordinated by AKAP4 (Luconi et al., 2011) raises the question of whether cAMP-responsive events associated with sperm capacitation are similarly affected by 4HNE adduction. In addition to the supporting data presented here on phosphorylation profiles and acrosome reaction rates, previous studies have also shown that exogenous 4HNE administration does compromise important correlates of the capacitation cascade including global increases in tyrosine phosphorylation (Baker et al., 2015) and downstream functional endpoints such as zona pellucida adhesion (Bromfield et al., 2015). However, as an important caveat in the interpretation of these data, we note that alternative elements of the motility apparatus (dynein, outer dense fiber protein 1) (Baker et al., 2015), capacitation signaling (PKA) (Baker et al., 2015), and membrane remodeling proteins (heat shock protein A2) (Bromfield et al., 2015) have been validated as primary targets of 4HNE-mediated modification. Such pervasive broad-spectrum effects make it extremely challenging to disentangle whether individual sperm proteins such as AKAP4 play a dominant role in the pathophysiological responses to 4HNE.

Of concern, these pleiotropic effects are elicited at concentrations of 4HNE that are well within the range attained under conditions of oxidative stress, which can reach as high as 5 mM (Uchida, 2003). They also proved refractory to a lipoxygenase inhibition strategy designed to disrupt the positive feedback loop of lipid peroxidation and hence prevent the induction of redox cycling cascades. In somatic and male germ cells alike, 4HNE adducted proteins are commonly targeted for proteolysis in order to mitigate the risk they pose to cellular homeostasis (Grune et al., 1995; Shang et al., 2001; Carbone et al., 2004b; Bromfield et al., 2017a). Here, we confirm that, despite differences in their solubility and putative interaction networks (Nipper et al., 2006), the innate stability of both proAKAP4 and AKAP4 is also significantly impacted by 4HNE adduction. Curiously however, our data do not support the mobilization of conventional proteolytic degradation pathways in the clearance of proAKAP4 and AKAP4, neither of which were rescued by pharmacological interventions intended to suppress proteasomal activity. Similarly, incubation of mature spermatozoa with GSH post-4HNE treatment also failed to rescue proAKAP4 and AKAP4 levels. A possible explanation for this response lies in the chemistry of 4HNE, which has the potential to form either Michael addition to thiol or amino compounds (via the C3 of the C2=C3 double bond) or Schiff bases (between the C1 carbonyl group and primary amines) (Dalleau et al., 2013). Notably, the kinetics of the Schiff base formation are inherently slow and reversible, whereas those of the Michael-adducts are more rapid and stable; hence Michael-adducts predominate (Dalleau et al., 2013). Although retro-Michael cleavage can occur, leading to resolution of 4HNE adducts, this is by no means a universal phenomenon and is instead heavily influenced by the context of the cellular environment in which this response is measured (Schaur et al., 2015). Irrespective, at present we remain uncertain what mechanism(s) may account for the loss of AKAP4 expression, however, we do not consider this an artifact caused by 4HNE adducts masking antibody recognition motifs. Indeed, we were able to demonstrate anti-proAKAP4 and anti-AKAP4 antibody recognition of 4HNE-treated recombinant AKAP4 protein. Moreover, these antibodies also readily labeled the residual proAKAP4 and AKAP4 *in situ* post-4HNE treatment, a setting in which the proteins harbored signatures of 4HNE adduction and aggregation. These latter findings are supported by independent evidence that heavily oxidized proteins resisting degradation have a propensity to crosslink and aggregate (Castro et al., 2017). Indeed, evidence from the somatic cell literature, has established that 4HNE oxidative modifications can inactivate target proteins by rendering them prone to formation of oligomers; both in solution and in the intracellular environment (Maniti et al., 2015). Accordingly, we have also shown that 4HNE treatment can result in significant accumulation of cytosolic protein deposits in the male germline (Cafe et al., 2019). We therefore, hypothesize that 4HNE induced aggregation of the proAKAP4 and AKAP4 proteins could reduce their activity and the efficacy with which they are recovered from cell lysates; although this prospect awaits further investigation.

## Conclusion

Here, we have confirmed the vulnerability of both proAKAP4 and AKAP4 to 4HNE modification throughout male germ cell development. Such modification leads to a range of adverse sequela that not only affect proAKAP4 and AKAP4 stability, but also compromises their functions in mature spermatozoa. These findings provide a physiological explanation for the loss of sperm motility and capacitation competence commonly encountered in response to oxidative stress and raise the prospect that such defects may help reduce the likelihood of sperm harboring oxidative DNA lesions from participating in fertilization. In this sense, oxidative damage of both proAKAP4 and AKAP4 may be considered a protective mechanism that limits the transmission of an altered male genome to the next generation. In any event, these data add to a growing body of literature emphasizing the need for novel therapeutic interventions to alleviate the burden of oxidative-stress mediated dysfunction in the male germline.

## Data Availability Statement

The raw data supporting the conclusions of this article will be made available by the authors, without undue reservation, to any qualified researcher.

## Ethics Statement

The studies involving human participants were reviewed and approved by The University of Newcastle Human Ethics Committee. All donors provided their written informed consent to participate in this study. The animal study was reviewed and approved by The University of Newcastle Animal Care and Ethics Committee.

## Author Contributions

BN conceived the study and wrote the first draft of the manuscript. IB, SC, AA, and NT were responsible for the study execution and data analysis. MD, NS, AE, TL, GD, MDD, and EB contributed to the conception and design of the study, and participated in the data analysis. All authors contributed to the manuscript revision, read, and approved the submitted version.

## Conflict of Interest

NS and MD are co-founders of SPQI – 4BioDx (Lille, France), a commercial company that markets the proAKAP4 and AKAP4 reagents used in this study. The remaining authors declare that the research was conducted in the absence of any commercial or financial relationships that could be construed as a potential conflict of interest.
