# Supplementary material for: A Kinase Anchor Protein 4 Is Vulnerable to Oxidative Adduction in Male Germ Cells
Source: Front Cell Dev Biol. 2019 Dec 20;7:319. doi: 10.3389/fcell.2019.00319 (PMC6933317; doi:10.3389/fcell.2019.00319)
Supplement: Supplementary file 1 [file Data_Sheet_1.PDF]

A Kinase Anchor Protein 4 is vulnerable to oxidative adduction in male germ cells

Brett Nixon, Ilana Bernstein, Shenae L. Cafe, Maryse Delehedde, Nicolas Sergeant, Andrew L. Eamens, Tessa Lord, Matthew D. Dun, Geoffry N. De Iuliis, and Elizabeth G. Bromfield

SUPPLEMENTARY MATERIAL

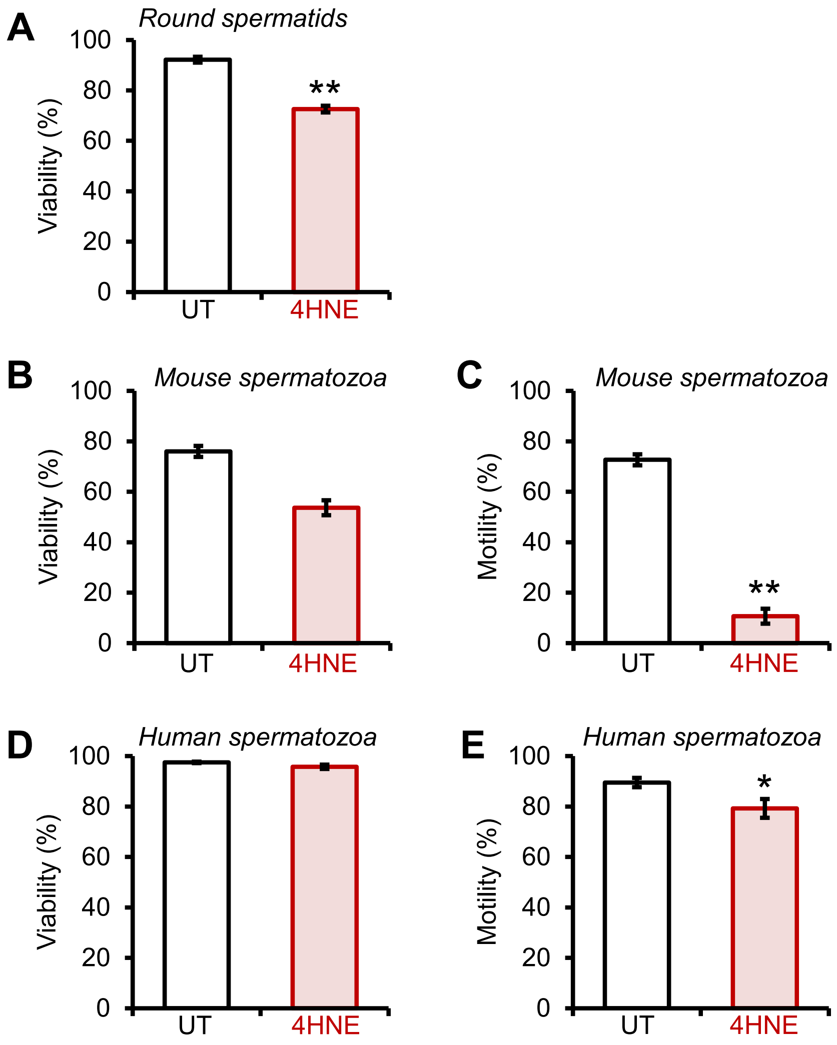

Supplementary Figure S1: Assessment of the impact of 4HNE treatment regimens on male germ cell viability and motility. Populations of (**A**) round spermatids, (**B,C**) mouse spermatozoa, and (**D,E**) human spermatozoa were incubated with the maximum dose of 4HNE used throughout this study (i.e. 100 μM) for 1 h alongside an untreated control (UT). Post-treatment, each population of cells was labeled with Eosin vitality stain, and assessed for the percentage of viable (unstained) cells. Alternatively, the motility of (**C**) mouse and (**E**) human spermatozoa was also assessed. In each instance, a minimum of 100 cells were scored across each of three replicates and data are presented as mean ± SEM. * $P < 0.05$, ** $P < 0.01$ relative to untreated control (UT).

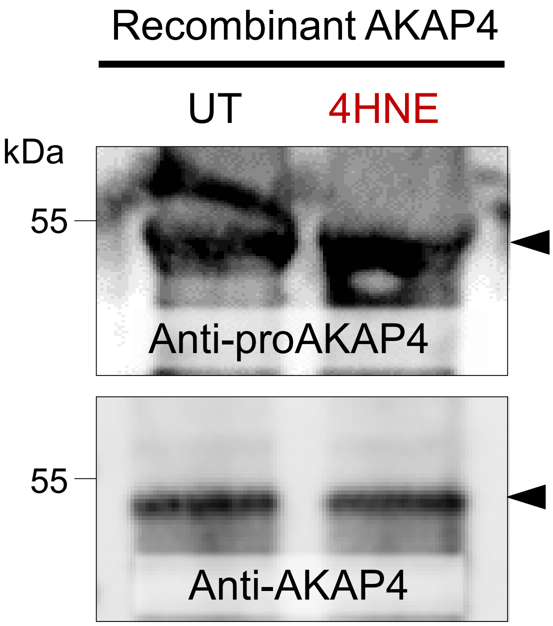

Supplementary Figure S2: Assessment of anti-proAKAP4 and anti-AKAP4 antibody affinity for 4HNE adducted recombinant AKAP4 protein. To control for the possibility of 4HNE-mediated masking of the epitopes recognized by either the anti-proAKAP4 or anti-AKAP4 antibodies, recombinant AKAP4 protein was treated with 4HNE under identical conditions to those imposed on germ cells (i.e. 50 µM 4HNE for 1 h at 37°C) prior to being prepared for immunoblotting. As shown, anti-proAKAP4 and anti-AKAP4 antibodies retained the ability to bind 4HNE-treated recombinant AKAP4 with comparable efficiency to that of the untreated control sample (UT). Arrowheads indicate protein bands corresponding to the expected size of recombinant AKAP4.

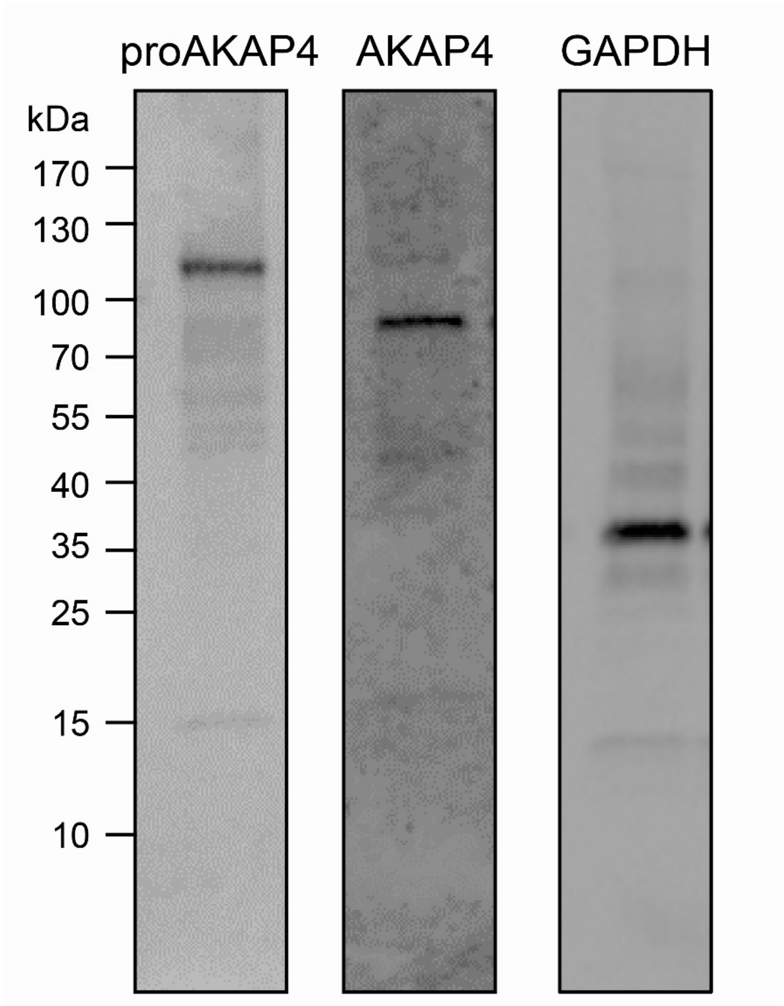

Supplementary Figure S3: Assessment of the specificity of anti-proAKAP4, anti-AKAP4, and anti-GAPDH antibodies used throughout this study. Notwithstanding some minor cross-reactivity, each of the antibodies used recognized predominant bands of the appropriate molecular weight for the target proteins.
